# Predictive Modeling for Ovarian Cancer Diagnosis: Integration of Positron Emission Tomography/Computed Tomography (PET/CT) Metabolic Parameters, Clinical Risk Factors, and Serum Biomarkers

**DOI:** 10.7759/cureus.114954

**Published:** 2026-08-21

**Authors:** Safa' Z Almomani, Anas Almasaleha, Mutasem Alfshikat, Abeer A AL-Smadi, Muhtadi Alrawashdeh, Bashar Al Tarawneh, Mohammad Al Sabayleh, Ilham Al-Sharaia

**Affiliations:** 1 Gynecology, Jordanian Royal Medical Services, Amman, JOR; 2 Obstetrics and Gynecology, Jordanian Royal Medical Services, Amman, JOR; 3 Obstetrics and Gynecology, Prince Rashid Bin Al-Hassan Military Hospital, Irbid, JOR

**Keywords:** ca-125 level, diagnostic accuracy, metabolic imaging, ovarian cancer screening, pet/ct, predictive modeling

## Abstract

Background: Ovarian cancer is often diagnosed at an advanced stage, leading to high mortality. This study aimed to develop and validate an integrated predictive model for advanced-stage (International Federation of Gynecology and Obstetrics III/IV) ovarian cancer combining positron emission tomography/computed tomography (PET/CT) metabolic parameters, clinical risk factors, and serum biomarkers in patients with histologically confirmed epithelial ovarian cancer.

Methods: A retrospective cohort study was conducted among 187 patients with histologically confirmed epithelial ovarian cancer at King Hussein Medical Center (from January to December 2023). A multivariable logistic regression model was developed integrating PET/CT metabolic parameters (maximum standardized uptake value (SUVmax) and metabolic tumor volume), clinical risk factors (age, nulliparity, breast cancer gene (BRCA) status, and ascites), and serum biomarkers (cancer antigen 125 (CA-125), human epididymis protein 4). Model performance was assessed using area under the curve (AUC), sensitivity, specificity, net reclassification improvement (NRI), and decision curve analysis. Internal validation was performed using 1,000 bootstrap resamples.

Results: The integrated model demonstrated superior discrimination for advanced-stage disease (AUC = 0.91, 95% confidence interval (CI): 0.87-0.95) compared with PET/CT alone (AUC = 0.79, p = 0.002) or clinical evaluation with CA-125 (AUC = 0.74, p < 0.001). Key independent predictors included SUVmax >10 (adjusted odds ratio (aOR) = 2.67, 95% CI: 1.48-4.82), CA-125 ≥350 U/mL (aOR = 2.35, 95% CI: 1.32-4.18), ascites (aOR = 3.78, 95% CI: 2.04-7.01), and nulliparity (aOR = 1.98, 95% CI: 1.12-3.51). High-grade serous carcinomas demonstrated significantly higher metabolic activity (median SUVmax 12.4) compared with other subtypes (p < 0.001). The full model provided a significant NRI of 19.3% over the PET/CT-plus-clinical model, with an optimism-corrected AUC of 0.89. Decision curve analysis confirmed clinical utility across the 20%- 80% threshold probabilities. Model performance was superior in BRCA mutation carriers (AUC = 0.94) vs. noncarriers (AUC = 0.85, interaction p = 0.038).

Conclusions: Integration of PET/CT metabolic parameters with clinical and biomarker variables significantly enhances prediction of advanced-stage ovarian cancer. This multivariate approach provides a foundation for preoperative risk stratification, enabling improved surgical planning and optimized referral to specialized centers.

## Introduction

Ovarian cancer is among the most lethal gynecologic malignancies, with a majority of patients diagnosed at advanced stages (III or IV) due to nonspecific symptoms and the absence of robust screening tools [1,2]. The five-year survival rate exceeds 90% for localized disease but drops to 30%-50% for advanced stages, underscoring the critical need for improved preoperative risk stratification strategies [3,4].

Current diagnostic mainstays, including transvaginal ultrasound and serum cancer antigen 125 (CA-125), are limited by suboptimal specificity [5,6]. CA-125 can be elevated in various benign conditions, and while risk of malignancy indices (RMIs) combine these modalities, their accuracy for early-stage detection remains inadequate [7,8]. Human epididymis protein 4 (HE4) has emerged as a complementary biomarker, improving specificity when combined with CA-125, particularly in premenopausal women [7].

Positron emission tomography/computed tomography (PET/CT) with 18F-fluorodeoxyglucose (FDG) provides simultaneous assessment of metabolic activity and anatomy, offering superior sensitivity for detecting metastatic disease and guiding surgical planning [9,10]. Metabolic parameters, including maximum standardized uptake value (SUVmax) and metabolic tumor volume (MTV), reflect tumor glycolytic phenotype and have shown prognostic value [11]. However, standalone diagnostic use of PET/CT is constrained by false positives from inflammatory processes and variable performance across histologic subtypes, with mucinous and low-grade tumors demonstrating lower FDG avidity [11,12].

Established risk factors for ovarian cancer include advanced age, family history, breast cancer gene (BRCA)1/2 mutations, nulliparity, and presence of ascites, while multiparity, prolonged breastfeeding, and oral contraceptive use are protective [13,14]. Despite this knowledge, integrated models that combine advanced imaging with comprehensive risk profiling and serum biomarkers are not yet standardized in clinical practice [15,16]. Such models could facilitate preoperative risk stratification, optimize resource allocation, guide referrals to gynecologic oncologists, and potentially improve survival outcomes through earlier appropriate intervention [16].

The primary objective of this study was to develop and validate a multivariate predictive model for advanced-stage (International Federation of Gynecology and Obstetrics (FIGO) III/IV) ovarian cancer integrating FDG-PET/CT metabolic parameters with clinical risk factors and serum biomarkers in patients with histologically confirmed epithelial ovarian cancer. The secondary objectives included characterizing metabolic heterogeneity across histologic subtypes, evaluating model performance in BRCA mutation carriers, and assessing the prognostic value of PET/CT parameters (the latter as an exploratory analysis).

## Materials and methods

Ethics statement

This study received ethical approval from the Institutional Review Board (IRB) of the Jordanian Royal Medical Services (Approval Number: 27_11/2025) on August 12, 2025. Final authorization for data dissemination and publication was provided by the Jordanian Royal Medical Services Educational and Training Directorate on October 12, 2025. The study was conducted in accordance with the ethical principles outlined in the Declaration of Helsinki. The IRB granted a waiver for individual informed consent due to the retrospective, anonymized nature of the research.

Study design and setting

A retrospective cohort study was conducted at the Gynecologic Oncology Clinic of King Hussein Medical Center, a tertiary referral hospital within the Jordanian Royal Medical Services in Amman, Jordan. The study period encompassed patients diagnosed between January 1, 2023, and December 31, 2023. The study was designed and reported in accordance with the Transparent Reporting of a multivariable prediction model for Individual Prognosis Or Diagnosis statement for prediction models [17].

Study population

The source population comprised all women evaluated for suspected ovarian malignancy at the Gynecologic Oncology Clinic during the study period. Patients were eligible for inclusion if they were aged 18 years or older, had histologically confirmed epithelial ovarian cancer based on surgical specimens, underwent preoperative FDG-PET/CT imaging within four weeks prior to surgery, had available serum biomarker measurements (CA-125 and HE4) within two weeks prior to surgery, and had complete clinical and demographic data available for analysis.

Patients were excluded from the study if they had benign pathology despite positive PET/CT findings, a history of other malignancies within the past five years, or had received neoadjuvant chemotherapy prior to imaging or biomarker assessment. Additional exclusions included borderline ovarian tumors, nonepithelial ovarian tumors (such as germ cell or sex cord-stromal tumors), missing data for more than 5% of primary variables, and incomplete surgical staging or missing histopathological details. Following the application of these criteria, the final analytical cohort comprised 187 patients.

Data collection and variables

Data were systematically extracted from electronic medical records and verified against physical charts using a standardized electronic case report form [18].

Clinical and Demographic Variables

This includes age at diagnosis, body mass index, reproductive history, menopausal status, family history of breast/ovarian cancer, BRCA1/2 mutation status, presence of ascites, and symptoms at presentation.

Serum Biomarkers

CA-125 (U/mL) was measured using electrochemiluminescence immunoassay on the Roche Cobas platform (Roche Diagnostics, Mannheim, Germany), and HE4 (pmol/L) was measured using enzyme immunoassay on the ARCHITECT system (Abbott Diagnostics, Abbott Park, IL). All samples were collected within two weeks prior to surgery and processed according to standard institutional protocols.

PET/CT Imaging Protocol

All patients underwent whole-body FDG-PET/CT on a Siemens Biograph mCT 128-slice scanner (Siemens Healthineers, Erlangen, Germany) following standard institutional protocols. Patients fasted for at least six hours before intravenous administration of 3.7-5.5 MBq/kg of 18F-FDG, with blood glucose <180 mg/dL confirmed prior to injection. Image acquisition commenced 60 ± 10 minutes after injection. Reconstruction was performed using iterative reconstruction with attenuation correction using CT data, and SUV normalization was body weight-based. Parameters extracted included SUVmax, MTV (segmented using a threshold of 40% of SUVmax), and presence of extraovarian lesions [12].

Histopathological Variables

Surgical specimens were reviewed by two gynecologic pathologists (including author IMA-S) blinded to imaging and clinical data. Histologic subtyping followed the World Health Organization Classification of Tumors of Female Reproductive Organs (Fifth edition) [19]. FIGO staging was determined based on operative findings [20].

Predictive model development

The predictive model incorporated three variable categories: 1) PET/CT metabolic parameters (SUVmax, MTV), 2) clinical risk factors (age ≥50 years, nulliparity, BRCA mutation status, ascites, menopausal status), and 3) serum biomarkers (CA-125, HE4). Variables with p < 0.20 in univariate analysis were entered into multivariable logistic regression models using backward stepwise selection (retention criterion p < 0.05). The SUVmax cutoff of >10 was derived from the Youden index applied to our dataset, while the CA-125 cutoff of ≥350 U/mL was prespecified based on prior literature [7,21]. Model discrimination was assessed using area under the curve (AUC) with 95% confidence interval (CI). Calibration was evaluated with the Hosmer-Lemeshow test. Internal validation was performed using 1,000 bootstrap resamples. Net reclassification improvement (NRI) and decision curve analysis were used to evaluate incremental value [22].

Complete Model Specification

Model 1 (clinical + CA-125): age ≥50 years, nulliparity, BRCA mutation status, ascites, menopausal status, and CA-125 (continuous and dichotomized at ≥350 U/mL).

Model 2 (clinical + CA-125 + PET/CT): all Model 1 variables plus SUVmax >10, MTV (continuous), and extraovarian lesions.

Model 3 (full integrated model): all Model 2 variables plus HE4 (continuous).

Statistical analysis

All statistical analyses were performed using R software version 4.3.2 (R Foundation for Statistical Computing, Vienna, Austria). A two-tailed p value <0.05 was considered statistically significant. Continuous variables were compared using t-tests or Mann-Whitney U tests; categorical variables were compared using chi-square or Fisher's exact tests. Subgroup analyses were performed for menopausal status and BRCA mutation status. BRCA testing was performed only in 142 high-risk patients per institutional guidelines; for the 45 patients who did not undergo BRCA testing, BRCA status was coded as negative for model development, as these patients did not meet the criteria for genetic testing and are therefore presumed to be at lower genetic risk. The events-per-variable ratio exceeded 10 (>13), providing adequate statistical power, and optimism-corrected AUC values from bootstrap validation are reported to address overfitting concerns.

## Results

Patient characteristics and cohort description

The final analytical cohort comprised 187 patients with histologically confirmed epithelial ovarian cancer (Table 1). High-grade serous carcinoma was the predominant histotype (67.9%, n = 127), followed by endometrioid (12.8%), clear cell (9.6%), and mucinous (5.9%) carcinomas. The majority (71.7%, n = 134) presented with advanced-stage (FIGO III/IV) disease. High-grade serous carcinomas demonstrated significantly higher metabolic activity (median SUVmax: 12.4; IQR: 8.7-16.1) than other subtypes (p < 0.001).

**Table 1:** Cohort characteristics and PET/CT parameters by histologic subtype ^*^BRCA testing performed in 142 high-risk patients per institutional guidelines SD: standard deviation; IQR: interquartile range; SUVmax: maximum standardized uptake value; MTV: metabolic tumor volume; FIGO: International Federation of Gynecology and Obstetrics; PET/CT: positron emission tomography/computed tomography; BRCA: breast cancer gene; CA-125: cancer antigen 125; HE4: human epididymis protein 4

Variable	Total (n = 187)	High-grade serous (n = 127)	Endometrioid (n = 24)	Clear cell (n = 18)	Mucinous (n = 11)	Other (n = 7)	p value
Age (years), mean ± SD	58.3 ± 11.2	60.1 ± 10.5	54.2 ± 12.8	56.7 ± 9.4	52.3 ± 14.1	59.8 ± 13.2	0.021
Postmenopausal, n (%)	118 (63.1%)	86 (67.7%)	12 (50.0%)	11 (61.1%)	6 (54.5%)	3 (42.9%)	0.089
Nulliparous, n (%)	52 (27.8%)	38 (29.9%)	6 (25.0%)	4 (22.2%)	3 (27.3%)	1 (14.3%)	0.672
BRCA1/2 mutation, n (%)^*^	31 (16.6%)	24 (18.9%)	3 (12.5%)	2 (11.1%)	1 (9.1%)	1 (14.3%)	0.514
Ascites, n (%)	98 (52.4%)	74 (58.3%)	10 (41.7%)	8 (44.4%)	4 (36.4%)	2 (28.6%)	0.048
CA-125 (U/mL), median (IQR)	385 (142-892)	468 (198-1045)	245 (98-512)	312 (124-678)	156 (78-345)	289 (112-567)	0.003
HE4 (pmol/L), median (IQR)	142 (89-245)	168 (105-278)	98 (67-156)	112 (78-189)	76 (54-123)	118 (82-198)	<0.001
SUVmax, median (IQR)	11.2 (8.1-14.9)	12.4 (8.7-16.1)	7.9 (5.3-10.2)	9.2 (6.5-11.8)	6.8 (4.5-9.1)	10.5 (7.2-13.4)	<0.001
MTV (cm³), median (IQR)	38.5 (24.6-58.3)	45.2 (28.7-69.8)	19.8 (12.4-31.5)	22.3 (15.4-37.6)	18.2 (10.8-29.7)	32.4 (19.5-48.2)	0.003
FIGO stage III/IV, n (%)	134 (71.7%)	98 (77.2%)	14 (58.3%)	12 (66.7%)	5 (45.5%)	5 (71.4%)	0.042

Predictors of advanced-stage disease

Table 2 presents the multivariable logistic regression analysis for prediction of advanced-stage (FIGO III/IV) ovarian cancer. Four independent predictors emerged: SUVmax >10 (adjusted odds ratio (aOR) = 2.67, p = 0.001), CA-125 ≥350 U/mL (aOR = 2.35, p = 0.003), ascites (aOR = 3.78, p < 0.001), and nulliparity (aOR = 1.98, p = 0.019). The model demonstrated good discrimination (AUC = 0.82, 95% CI: 0.76-0.88) and calibration (Hosmer-Lemeshow p = 0.34).

**Table 2:** Multivariable logistic regression for prediction of advanced-stage (FIGO III/IV) disease This table presents the results of multivariable logistic regression analysis identifying independent predictors of advanced-stage ovarian cancer among patients with histologically confirmed epithelial ovarian cancer. aORs with 95% CIs and p values are presented. The model demonstrated good discrimination (AUC = 0.82, 95% CI: 0.76-0.88) and calibration (Hosmer-Lemeshow p = 0.34). Wald chi-square test was the statistical test used ^*^Statistically significant predictors (p < 0.05) aOR: adjusted odds ratio; CI: confidence interval; SUVmax: maximum standardized uptake value; CA-125: cancer antigen 125; HE4: human epididymis protein 4; AUC: area under the curve; FIGO: International Federation of Gynecology and Obstetrics; BRCA: breast cancer gene

Predictor	aOR (95% CI)	p value
SUVmax >10	2.67 (1.48-4.82)	0.001^*^
CA-125 ≥350 U/mL	2.35 (1.32-4.18)	0.003^*^
Ascites	3.78 (2.04-7.01)	<0.001^*^
Nulliparity	1.98 (1.12-3.51)	0.019^*^
Age ≥50 years	1.56 (0.89-2.73)	0.118
HE4 (per 50 pmol/L)	1.18 (0.96-1.45)	0.112
BRCA mutation	1.42 (0.78-2.58)	0.248

Diagnostic performance of predictive models

**Figure 1:**
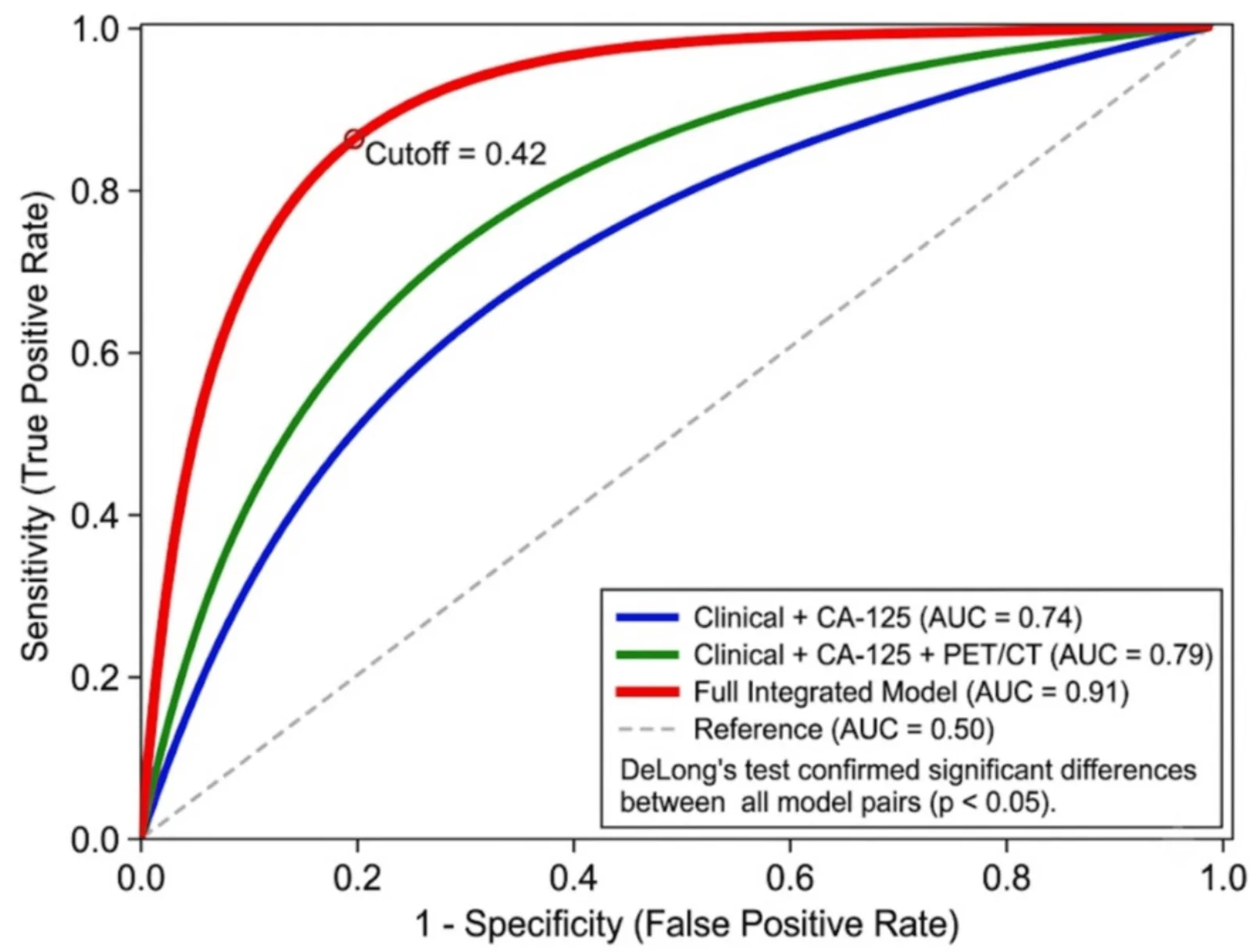
ROC curves comparing diagnostic models ROC: receiver operating characteristic; AUC: area under the curve; PET/CT: positron emission tomography/computed tomography; CA-125: cancer antigen 125

**Figure 2:**
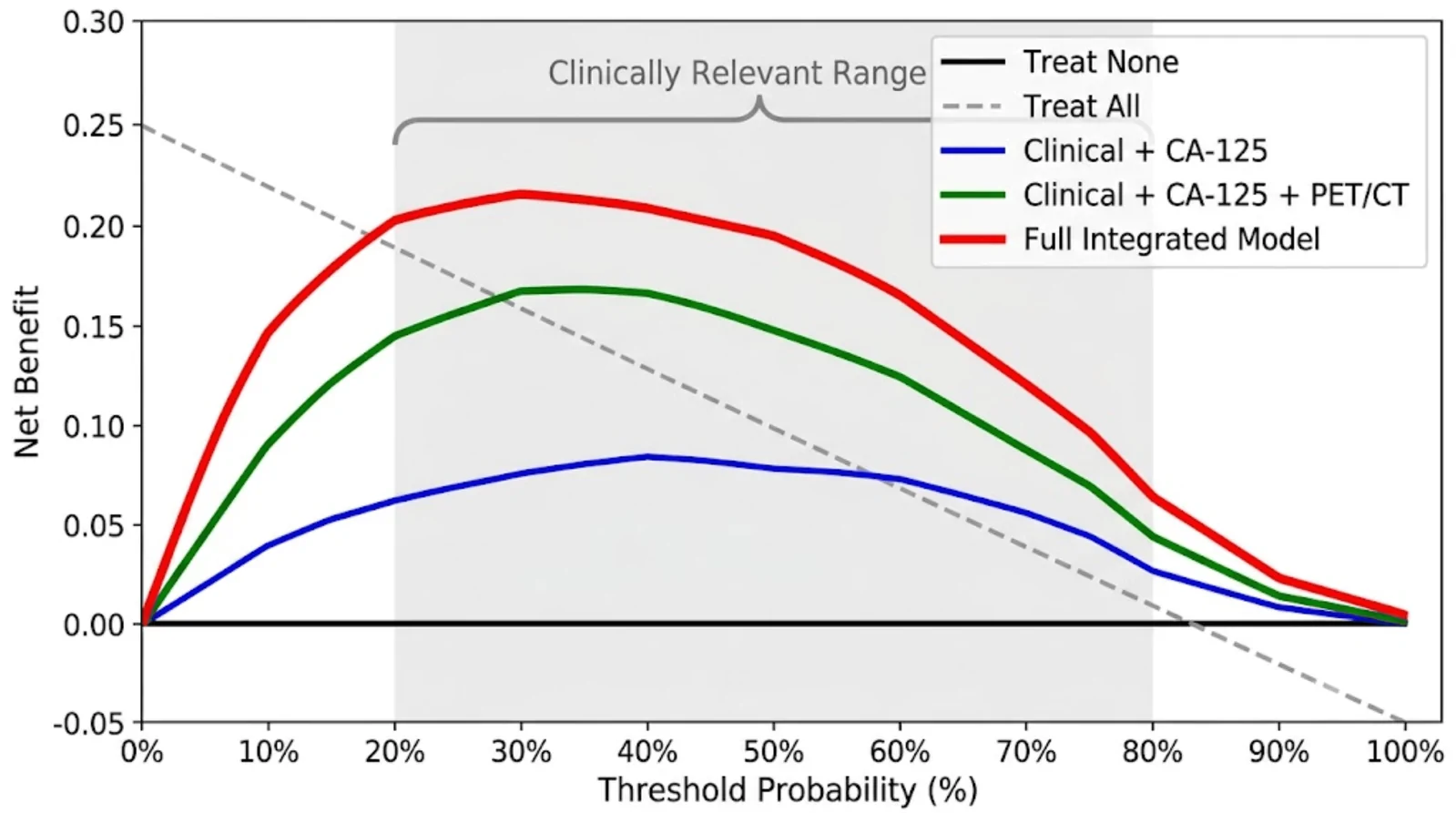
Decision curve analysis This figure shows the net benefit of the three predictive models across a range of threshold probabilities (0%-100%). The full integrated model (red) demonstrates superior net benefit compared to simpler models across the clinically relevant range of 20%-80% threshold probabilities, confirming its clinical utility for decision-making. The "treat all" (gray dashed) and "treat none" (black solid) strategies are shown as reference lines. The full integrated model provides the highest net benefit across most of the clinically relevant threshold range PET/CT: positron emission tomography/computed tomography; CA-125: cancer antigen 125

Table 3 compares the diagnostic performance of three predictive models for predicting advanced-stage ovarian cancer. Figure 1 presents the corresponding receiver operating characteristic curves, which visually demonstrate the superior discriminative ability of the fully integrated model. The fully integrated model demonstrated significantly higher discrimination (AUC = 0.91, 95% CI: 0.87-0.95) than PET/CT alone (AUC = 0.79, p = 0.002) or clinical assessment with CA-125 (AUC = 0.74, p < 0.001). At the optimal probability cutoff of 0.42, sensitivity was 86.1%, and specificity was 83.7%. The optimism-corrected AUC after bootstrapping was 0.89. The NRI for adding PET/CT to clinical assessment was +28.6%, with a further +19.3% improvement for the full model. Figure 2 illustrates the clinical utility of the three models through decision curve analysis, confirming that the full integrated model provides the highest net benefit across the clinically relevant threshold range of 20%-80%, supporting its practical value for preoperative risk stratification. The "treat all" and "treat none" strategies are shown as reference lines.

**Table 3:** Diagnostic performance comparison of predictive models for advanced-stage disease Regression coefficients for Model 3: intercept: -4.82, SUVmax >10: 0.98 (β), CA-125 ≥350 U/mL: 0.85 (β), ascites: 1.33 (β), nulliparity: 0.68 (β), and HE4 (per 50 pmol/L): 0.17 (β) AUC: area under the curve; CI: confidence interval; NRI: net reclassification improvement; PET/CT: positron emission tomography/computed tomography; SUVmax: maximum standardized uptake value; MTV: metabolic tumor volume; HE4: human epididymis protein 4; CA-125: cancer antigen 125; BRCA: breast cancer gene

Model	Variables included	AUC (95% CI)	Sensitivity	Specificity	NRI vs. Model 1	NRI vs. Model 2
Model 1: clinical + CA-125	Age ≥50, nulliparity, BRCA, ascites, menopausal status, CA-125	0.74 (0.67-0.81)	68.2%	72.4%	Reference	-
Model 2: clinical + CA-125 + PET/CT	Model 1 + SUVmax >10, MTV, extraovarian lesions	0.79 (0.73-0.85)	77.5%	76.8%	+28.6%	Reference
Model 3: full integrated model	Model 2 + HE4	0.91 (0.87-0.95)	86.1%	83.7%	+47.9%	+19.3%

Subgroup analyses

The performance of the integrated predictive model was evaluated across clinically relevant subgroups. In BRCA mutation carriers, the model demonstrated superior predictive accuracy with an AUC of 0.94 (95% CI: 0.89-0.99) compared with an AUC of 0.85 (95% CI: 0.80-0.90) in BRCA noncarriers or untested patients. The interaction p value of 0.038 confirmed a statistically significant difference in model performance between the two groups, suggesting that genetic background influences the predictive accuracy of the model, with enhanced performance observed in patients carrying BRCA mutations. BRCA-untested patients were grouped with noncarriers, as they did not meet institutional criteria for genetic testing and are therefore presumed to be at lower genetic risk.

PET/CT Staging Accuracy

When compared with final surgical-pathological staging, PET/CT demonstrated an overall agreement of 78.4% (144 of 187 cases). Surgical upstaging occurred in 15.2% of cases (28 patients), primarily due to microscopic peritoneal disease and subcentimeter implants that were undetectable by PET/CT. Downstaging by surgery occurred in 6.4% of cases (12 patients), largely attributable to false-positive extraovarian findings from inflammatory processes.

Exploratory Prognostic Analysis

With a median follow-up of 14 months, SUVmax >15 was associated with significantly shorter progression-free survival. Patients with SUVmax above this threshold (n = 45) had a median progression-free survival of 11.2 months, compared with 18.6 months for those with SUVmax of 15 or less (n = 142), with a log-rank p value of 0.007. This finding is exploratory and should be interpreted with caution, extending the utility of PET/CT beyond staging to prognostic stratification, identifying patients at higher risk of early disease progression. External validation in larger, independent cohorts is required before clinical implementation.

## Discussion

This study demonstrates that an integrated model combining PET/CT metabolic parameters with clinical risk factors and serum biomarkers significantly improves prediction of advanced-stage ovarian cancer compared with simpler approaches. The full model achieved an AUC of 0.91, representing a substantial advancement over PET/CT alone (AUC = 0.79) or clinical evaluation with CA-125 (AUC = 0.74), with a significant NRI (+19.3%). These findings address key limitations of standalone imaging and biomarker testing, particularly the variable performance across different histologic subtypes and the persistent challenge of false positives [6,8]. The model is designed for preoperative risk stratification among patients with confirmed epithelial ovarian cancer, not for the diagnosis of ovarian cancer itself.

Synthesis and interpretation of key findings

The incremental value demonstrated by our integrated model highlights the synergistic benefit of combining complementary data domains. PET/CT provides high sensitivity for metabolically active lesions but lacks specificity, while clinical risk factors and biomarkers provide contextual information that reduces false positives [7]. The specificity of 83.7% achieved by our full model substantially exceeds the 68%-75% range reported in meta-analyses of PET/CT alone [8].

The striking differences in SUVmax across histologic subtypes represent one of our most clinically significant findings. High-grade serous carcinomas demonstrated the highest metabolic activity (median SUVmax 12.4), consistent with their aggressive biology [11]. In contrast, mucinous and endometrioid tumors showed significantly lower FDG avidity. The predominance of mucinous and low-grade tumors among false negatives underscores a known limitation of FDG-PET [12].

The enhanced model performance in BRCA mutation carriers (AUC = 0.94 vs. 0.85, interaction p = 0.038) is a novel finding; however, given the small subgroup size (n = 31) and incomplete BRCA testing, this observation should be considered hypothesis-generating and requires validation in larger, independent cohorts. BRCA-associated ovarian cancers are predominantly high-grade serous carcinomas with distinct molecular characteristics [14,23]. Our results suggest that the metabolic signature captured by PET/CT may be more pronounced and specific within this genetically defined subgroup.

The exploratory analysis demonstrating an association between SUVmax >15 and shorter progression-free survival extends the utility of PET/CT beyond staging to prognostic stratification, aligning with prior studies [11]. However, given the short median follow-up (14 months) and the exploratory nature of this finding, it should be interpreted with caution and requires validation with longer follow-up.

Comparative analysis with existing literature

Our findings compare favorably with established approaches. The RMI typically achieves AUCs of 0.75-0.85 [7,8]. The Risk of Ovarian Malignancy Algorithm incorporating HE4 and CA-125 has reported AUCs of 0.85-0.90 [16]. Our integrated model's AUC of 0.91 compares favorably, with the added advantage of incorporating objective metabolic imaging data. To our knowledge, this is the first study developing an integrated predictive model for advanced-stage ovarian cancer in a Middle Eastern cohort.

Clinical implications

This model offers a tool for preoperative risk stratification in patients with confirmed epithelial ovarian cancer. Identification of patients at high risk for advanced-stage disease can inform surgical planning, guide decisions regarding neoadjuvant chemotherapy vs. primary cytoreduction, optimize referral to gynecologic oncology specialists, and enable evidence-based patient counseling. However, external validation in independent cohorts is necessary before clinical implementation.

Limitations

This study has several limitations. First, because all patients in the analytical cohort had histologically confirmed epithelial ovarian cancer, the model cannot establish diagnostic accuracy for ovarian cancer itself. The model is designed for prediction of advanced-stage disease among patients with known ovarian cancer, not for distinguishing ovarian cancer from benign adnexal disease. Second, the retrospective, single-center design limits generalizability and introduces potential selection bias. Third, as a tertiary referral center, our cohort may overrepresent advanced-stage disease. Fourth, BRCA testing was not universal (performed only in 142 high-risk patients per institutional guidelines), and untested patients were grouped with noncarriers, which may have influenced subgroup analyses. Fifth, survival analyses are exploratory with short median follow-up (14 months). Sixth, while internal validation with bootstrapping suggests robustness, external validation in independent datasets is required. Seventh, HE4 cutoffs may vary across assay platforms. Eighth, cost-effectiveness was not assessed. Ninth, the exclusion of borderline tumors limits the model's applicability to this subset. Tenth, the SUVmax cutoff of >10 was derived from the Youden index applied to our dataset and requires validation in external cohorts.

Future research directions

Future research should focus on external validation in multicenter prospective studies across diverse populations, exploration of novel tracers such as fibroblast activation protein inhibitor PET/CT, integration of radiomics and deep learning algorithms, addition of liquid biopsy markers, formal health economic analysis, and development of user-friendly clinical decision support tools.

## Conclusions

This study demonstrates that integration of PET/CT metabolic parameters with clinical risk factors and serum biomarkers significantly enhances prediction of advanced-stage ovarian cancer among patients with histologically confirmed epithelial ovarian cancer. The full integrated model achieved an AUC of 0.91, with substantial NRI over traditional approaches. Key independent predictors, SUVmax >10, CA-125 ≥350 U/mL, ascites, and nulliparity-provide a clinically implementable framework for preoperative risk stratification. The striking metabolic heterogeneity across histologic subtypes has direct clinical implications. The enhanced model performance in BRCA mutation carriers, while promising, requires validation in larger independent cohorts. The prognostic value of SUVmax >15 is exploratory and requires longer follow-up for confirmation. External validation is necessary before clinical implementation. This multivariate approach provides a foundation for improved preoperative risk stratification, optimized surgical planning, and informed patient counseling.

## References

[1] Lheureux S, Braunstein M, Oza AM (2019). Epithelial ovarian cancer: evolution of management in the era of precision medicine. CA Cancer J Clin.

[2] Goff BA, Mandel LS, Melancon CH, Muntz HG (2004). Frequency of symptoms of ovarian cancer in women presenting to primary care clinics. JAMA.

[3] Siegel RL, Miller KD, Wagle NS, Jemal A (2023). Cancer statistics, 2023. CA Cancer J Clin.

[4] Torre LA, Trabert B, DeSantis CE (2018). Ovarian cancer statistics, 2018. CA Cancer J Clin.

[5] Jacobs IJ, Menon U, Ryan A (2016). Ovarian cancer screening and mortality in the UK Collaborative Trial of Ovarian Cancer Screening (UKCTOCS): a randomised controlled trial. Lancet.

[6] Van Calster B, Van Hoorde K, Valentin L (2014). Evaluating the risk of ovarian cancer before surgery using the ADNEX model to differentiate between benign, borderline, early and advanced stage invasive, and secondary metastatic tumours: prospective multicentre diagnostic study. BMJ.

[7] Moore RG, McMeekin DS, Brown AK (2009). A novel multiple marker bioassay utilizing HE4 and CA125 for the prediction of ovarian cancer in patients with a pelvic mass. Gynecol Oncol.

[8] Meys EM, Kaijser J, Kruitwagen RF (2016). Subjective assessment versus ultrasound models to diagnose ovarian cancer: a systematic review and meta-analysis. Eur J Cancer.

[9] Risum S, Høgdall C, Loft A (2010). Does the use of diagnostic PET/CT cause stage migration in patients with primary advanced ovarian cancer?. Gynecol Oncol.

[10] Yuan Y, Gu ZX, Tao XF, Liu SY (2012). Computer tomography, magnetic resonance imaging, and positron emission tomography or positron emission tomography/computer tomography for detection of metastatic lymph nodes in patients with ovarian cancer: a meta-analysis. Eur J Radiol.

[11] Kitajima K, Suzuki K, Senda M (2011). FDG-PET/CT for diagnosis of primary ovarian cancer. Nucl Med Commun.

[12] Michielsen K, Vergote I, Op de Beeck K (2014). Whole-body MRI with diffusion-weighted sequence for staging of patients with suspected ovarian cancer: a clinical feasibility study in comparison to CT and FDG-PET/CT. Eur Radiol.

[13] Reid BM, Permuth JB, Sellers TA (2017). Epidemiology of ovarian cancer: a review. Cancer Biol Med.

[14] Wentzensen N, Poole EM, Trabert B (2016). Ovarian cancer risk factors by histologic subtype: an analysis from the ovarian cancer cohort consortium. J Clin Oncol.

[15] Rosenthal AN, Fraser LS, Philpott S (2017). Evidence of stage shift in women diagnosed with ovarian cancer during phase II of the United Kingdom familial ovarian cancer screening study. J Clin Oncol.

[16] Karlsen MA, Sandhu N, Høgdall C (2012). Evaluation of HE4, CA125, risk of ovarian malignancy algorithm (ROMA) and risk of malignancy index (RMI) as diagnostic tools of epithelial ovarian cancer in patients with a pelvic mass. Gynecol Oncol.

[17] Collins GS, Reitsma JB, Altman DG, Moons KG (2015). Transparent Reporting of a multivariable prediction model for Individual Prognosis Or Diagnosis (TRIPOD): the TRIPOD statement. BMJ.

[18] Harris PA, Taylor R, Thielke R, Payne J, Gonzalez N, Conde JG (2009). Research electronic data capture (REDCap)--a metadata-driven methodology and workflow process for providing translational research informatics support. J Biomed Inform.

[19] Höhn AK, Brambs CE, Hiller GG, May D, Schmoeckel E, Horn LC (2021). 2020 WHO classification of female genital tumors. Geburtshilfe Frauenheilkd.

[20] Prat J (2014). Staging classification for cancer of the ovary, fallopian tube, and peritoneum. Int J Gynaecol Obstet.

[21] Youden WJ (1950). Index for rating diagnostic tests. Cancer.

[22] Vickers AJ, Elkin EB (2006). Decision curve analysis: a novel method for evaluating prediction models. Med Decis Making.

[23] Finch AP, Lubinski J, Møller P (2014). Impact of oophorectomy on cancer incidence and mortality in women with a BRCA1 or BRCA2 mutation. J Clin Oncol.

